# Toward a paradigm shift from deficit-based to proactive speech and language treatment: Randomized pilot trial of the Babble Boot Camp in infants with classic galactosemia

**DOI:** 10.12688/f1000research.18062.5

**Published:** 2020-07-06

**Authors:** Beate Peter, Nancy Potter, Jennifer Davis, Inbal Donenfeld-Peled, Lizbeth Finestack, Carol Stoel-Gammon, Kari Lien, Laurel Bruce, Caitlin Vose, Linda Eng, Hanako Yokoyama, Daniel Olds, Mark VanDam

**Affiliations:** 1 Speech and Hearing Science, College of Health Solutions, Arizona State University, Tempe, AZ, USA; 2 Department of Communication Sciences and Disorders, Saint Louis University, Saint Louis, MO, USA; 3 Department of Speech and Hearing Sciences, Elson S. Floyd College of Medicine, Washington State University, Spokane, WA, USA; 4 Department of Speech-Language-Hearing Services, University of Minnesota, Minneapolis, MN, USA; 5 Department of Speech and Hearing Sciences, University of Washington, Seattle, WA, USA; 6 Department of Communication Sciences and Disorders, Syracuse University, Syracuse, NY, USA

**Keywords:** speech disorder, language impairment, genetic risk, infant, very early intervention, prevention

## Abstract

**Background:** Speech and language therapy is typically initiated reactively after a child shows delays. Infants with classic galactosemia (CG), a metabolic disease with a known high risk for both speech and language disorders, hold the keys towards evaluating whether preventive treatment is effective when the risks are known at birth. We present pilot data from a randomized parallel trial of an innovative proactive speech and language intervention program, the Babble Boot Camp (BBC).

**Method**: Five children with CG, otherwise healthy, participated in the study from approximately 2 to 24 months of age. One of these was randomly selected as control receiving conventional management, which typically starts at age 2-3 years. A pediatric speech-language pathologist met weekly via telepractice with the parents in the treatment cohort. Parents implemented the prespeech, speech, and language stimulation and expansion activities according to the intervention protocol. The control child was still too young for conventional treatment. Primary outcome measures were speech sound production complexity in babble and speech and expressive vocabulary size. Secondary outcome measures were vocalization rates and developmental milestones in communication, motor, and cognition. The trial is ongoing.

**Results**:  All four treated children had higher speech sound skills in babble, three had higher speech sound skills in meaningful speech, two had higher expressive vocabularies, three had higher global developmental scores, and two had higher vocalization rates, compared to the control child with CG.

**Discussion:** Given the high risk for speech and language delays in children with CG, finding on-schedule abilities in two or more of the treated children but not the untreated child is unexpected under random conditions. The trends toward beneficial effects of the BBC on speech sound production, expressive language, and communication milestones warrant appropriately powered larger clinical trials with full randomization.

**Trial registration: **ClinicalTrials.gov NCT03838016 (12 ^th^ February 2019).

## Introduction

Difficulties with speech and language are common among young children. In the US, 11% of children age 3 to 6 years have a communication disorder ( Black *et al.*, 2015). Many parents who are concerned about their child’s ability to talk ask the child’s doctor, who, in turn, may refer them to a speech-language pathologist. By the time a referral is made, the child may already be two, three, or even four years old and has passed critical stages in the process of speech and language development ( Cates *et al.*, 2012). There is strong evidence that early interventions for children with known risks or first signs of a variety of disorders are highly effective, for instance interventions for children with autism spectrum disorder as young as 12 months ( Dawson *et al.*, 2010; Guralnick, 2011; Rogers *et al.*, 2014). However, very early speech and language services are not yet available, in part because speech and language are later-developing skills and disorders in these areas cannot be reliably diagnosed on behavioral grounds until an age when deficits become evident, which is greater than 24 months for speech ( Goldman & Fristoe, 2015) and greater than 36 months for language ( Semel *et al.*, 2004).

There are some early red flags during infancy that may foreshadow a later diagnosis of speech sound disorder and language impairment. Examples are sparse and delayed babbling ( American Speech-Language-Hearing Association, 2007; DePaolis *et al.*, 2013; McCune & Vihman, 2001; Oller *et al.*, 1998; Oller *et al.*, 1999; Stoel-Gammon, 1989; Stoel-Gammon, 2011; Vihman & Greenlee, 1987; Vihman *et al.*, 1985), low engagement in activities with joint attention ( Mundy *et al.*, 2007), and failure to use gestures ( Rowe & Goldin-Meadow, 2009). Specifically regarding babbling, a close predictive link between quantity/quality of babble and speech and language development later on has been previously described in typical children ( American Speech-Language-Hearing Association, 2007; DePaolis *et al.*, 2013; McCune & Vihman, 2001; Oller *et al.*, 1998; Oller *et al.*, 1999; Stoel-Gammon, 1989; Stoel-Gammon, 2011; Vihman & Greenlee, 1987; Vihman *et al.*, 1985).

Previous studies have investigated the effect of training parents to engage in activities designed to foster language growth in their children, both regarding typically developing children ( Roberts *et al.*, 2014; Tomasello & Farrar, 1986; Tomasello & Kruger, 1992) and children with autism spectrum disorder, Down syndrome, intellectual disability, and other conditions that are known to affect language development ( Roberts & Kaiser, 2011; Wetherby *et al*., 2014). In terms of caregiver training strategies, one intervention study via parent training demonstrated that a four-step approach consisting of teaching, modeling, coaching, and reviewing was effective toward increasing children’s expressive language skills at ages 24 to 42 months ( Roberts *et al*., 2014). Another approach was based on a six-step strategy consisting of (a) initiation and joint planning, (b) observation, (c) action (modeling and practice), (d) feedback, (e) reflection, and (f) evaluation, described for use with a child age 26 months ( Akamoglu & Dinnebeil, 2017). However, no study targeting prelinguistic skills included children younger than 12 months. Whether treatment focusing on earliest signals of communication has a beneficial effect on later speech and language development is unknown because such treatments have not been developed and validated. Going even further back in the developmental trajectory, some children are born with genetic or other risk factors for speech and language disorders; this risk is known long before prespeech behaviors such as coo and babble and actual speech and language emerge. The question is whether proactive, preventive treatment, if it existed, could reduce the deleterious effects associated with the risk factors in these cases and thereby improve outcomes.

Infants with classic galactosemia (CG) are an ideal population to investigate whether proactive interventions during the first two years of life, long before traditional assessment and intervention are available, can significantly improve speech and language outcomes. CG is a recessively inherited inborn error of metabolism diagnosed via newborn screening, with incidence rates in the US ranging from 1/30,000 to 1/60,000. Worldwide, incidence rates are highest among Caucasians, especially individuals of Irish descent ( Coss *et al*., 2013; Jumbo-Lucioni *et al*., 2012). Newborn diagnosis can be life-saving because of the deleterious effects of galactose buildup in the child’s blood that can occur if dietary restrictions are not implemented immediately. Despite rigorous dietary management, however, children with this disease have a substantially higher risk, compared to the typically developing (TD) population. These risk conditions include motor and learning disabilities ( Antshel *et al.*, 2004; Karadag *et al.*, 2013; Potter *et al.*, 2013) but also, importantly, severe speech and language disorders. Speech disorders were reported in 77% of children with CG ( Hughes *et al.*, 2009), compared to 3.8% among children generally ( Shriberg *et al.*, 1999), and language impairment in roughly 56% to 71% of children with CG (73% to 92% of children with CG who also had speech disorders ( Waggoner *et al.*, 1990) ( Potter *et al.*, 2008)), compared to 7.4% among children generally ( Tomblin *et al.*, 1997). This elevated risk, coupled with the early identification, makes children with CG an ideal population in which to examine the efficacy of prospective intervention therapy. If proactive intervention is shown to be more effective than conventional management, this has the potential to change the management model from deficit-based to preventive services for these infants. It will also motivate similar studies in infants with other types of risk for communication disorders, for instance very low birth weight and 7q11.23 duplication syndrome. That is, children known to be at risk may benefit from early, prospective intervention, thus improving outcomes.

The Babble Boot Camp (BBC)© is a program of activities and routines designed for infants and toddlers during the pre-speech and very early speech and language stages. It contains components intended to shape dyadic interactions across modalities, stimulate earliest vocalizations (coo, babble), support emergence of first words and sentences, and foster vocabulary and syntax growth. The active phase of intervention covers ages 2 to 24 months, with plans for follow-up testing using a professional evaluation of speech, language, and cognitive abilities at ages 30, 42, and 54 months. This program is unique in that it leverages knowledge of genetic risks for speech and language difficulties that are identified at birth via a diagnosis of CG, begins with infants as young as age 2 months, and addresses signals of communication starting with eye contact and pre-babble vocalizations, then progresses through all other prelinguistic and linguistic stages until age 24 months.

Here, we report pilot results of the BBC. This Phase 0 exploratory study demonstrates, with clear clinical application, a viable proactive early intervention approach for minimizing speech and language disorders in a vulnerable population of infants with a known genetic risk for these disorders. The purpose of this pilot study was to demonstrate feasibility and to conduct initial comparisons between children with CG participating in the BBC treatment and the control child with CG regarding developmental areas of interest. The primary focus was speech and language development measured with standardized assessments, with secondary attention to cognitive and motor development.

## Methods

This study was conducted with approval of the Institutional Review Boards at Arizona State University (IRB ID # STUDY00004969) and Washington State University (IRB ID # 13099). The design is a randomized parallel trial. The study began on January 31, 2017 and is ongoing. Parents learned about the study through online research announcements and referrals from physicians and other service providers, then contacted the research team. Once eligibility for participation was established and parents made the decision to participate, they gave written permission for their infants’ participation and written consent for their own participation. The study is listed on ClinicalTrials.gov under NCT03838016. The Babble Boot Camp is copyrighted and listed at Arizona State University under Technology ID M19-186L.

### Participants

The current participants are 25 children with CG and their parents. Here, we report on a subset of the children, the five oldest children with the longest participation record, for whom a nearly complete dataset is available through age 24 months. This pilot treatment cohort consists of four children, two girls (codes CG1, CG2) and two boys (CG4, CG5). Note that CG1 only participated in the study up to age 18 months, due to personal circumstances. One additional boy with CG was randomly selected to serve as a control who did not participate in the BBC treatment program. All families participated in the close monitoring components of the study, described further below. For purposes of comparison to typical children, archival data from test norms and publications, described below, were used. In the near future, the parallel design will be built out by recruiting more children with CG into the treatment and control cohorts and also by creating a control cohort consisting of children developing typically. During that next phase of the project, randomization and blinding will be used, the latter of which can only be applied to research team members who analyze the data.

In- and exclusionary criteria are identical for the treatment and control children with CG and are designed to evaluate the effects of the treatment while keeping all other factors the same. Inclusionary criteria are the following: Age at entry into the study is approximately 2 to 4 months. All infants are required to have a newborn diagnosis of CG. Boys and girls of any racial/ethnic background are equally eligible to participate, but given the highest CG prevalence rate among Caucasians, proportionally high numbers of Caucasians are expected. At least one parent or caregiver must enroll as a participant because we also collect quality of life information from the adults. The adults who enroll can be biological parents, adoptive parents, foster parents, or regular caregivers who are not related to the child as long as they provide care to the child on a regular basis. Primary language in the home must be English and at least one parent or caregiver must have at least an 8 ^th^ grade education. Because the intervention is implemented via telepractice software, any family whose primary language is English can participate, regardless of country of residence. Exclusionary criteria are the following: Galactosemia types other than CG; medical or sensory diagnosis that could introduce confounding, e.g., Trisomy 21 or deafness. Note that one child with CG, enrolled into the BBC as an infant, met the original criteria but later developed chronic ear infections requiring surgical insertion of pressure equalizing tubes at age 17 months; at the same time, she also underwent a frenectomy to address a tongue tie. Due to concerns regarding her hearing acuity and restricted tongue movement prior to these surgeries, her data are excluded from this report. Additionally, as noted in the consent form, families would be removed from the study if they missed more than half of their weekly meetings with the SLP over a two-month period and did not respond to re-engagement efforts. This was not the case for any of the children described in this study.

All five children live at home with both biological parents and are cared for during the day by their mothers while their fathers are at work. For each of these five families, parental levels of education, an estimate of socio-economic status, include at least some college (for details, see Table 1 below). Consistent with the high CG prevalence rates among Caucasians worldwide, all five children are Caucasian, and all reside in the US. Although additional interventions beyond the BBC, where available, are allowed, none of the children described here were receiving any additional services.

**Table 1.** Demographic information and fidelity of parent follow-through regarding weekly SLP sessions, weekly home videos, and monthly audio recordings.

Code	Sex	Highest Level Education Mother	Highest Level Education Father	% SLP Sessions Attended	% Weekly Videos Sent to SLP	% Monthly Audio Recordings Sent to Research Team
CG1	Girl	Some college	Bachelor’s	68.89	70.67	91.67
CG2	Girl	Bachelor’s	Graduate	93.33	65.77	68.18
CG4	Boy	Associate’s	Associate’s	97.83	65.77	95.24
CG5	Boy	Bachelor’s	Bachelor’s	97.53	77.38	94.74
CGCTR1	Boy	Associate’s	Bachelor’s	N/A	N/A	82.35

### Materials and procedures

The BBC is implemented via parent training by a speech-language pathologist (SLP) with expertise in early childhood development and earliest signals of communication. This SLP implements the program in all families in the treatment cohort. She uses a HIPAA-compliant telepractice computer interface to connect with the families. Parents learn about the typical milestones of prespeech, speech, and language development and potential red flags for delays, and, importantly, they are introduced to specific activities and routines that are designed to support typical development for all stages of the program and development beyond.

The program is built around 17 activities and routines designed to foster relevant communication skills for children ages 2 to 24 months. A description of these activities and routines is available at the Open Science Framework entry for the Babble Boot Camp ( https://osf.io/yzht4/). Activities are components of the program that take place for at least 5 minutes per day, whereas routines are designed to become daily habits in the parent-child interactions. Examples of activities are stimulating and reinforcing babble by showing the child videos of babbling babies and enriching the child’s linguistic environment with joint book reading ( Storkel *et al.*, 2017). Examples of routines are saying the names of objects that the child points to ( Dimitrova *et al.*, 2016), and expanding child utterances to provide slightly more complex model sentences ( Hassink & Leonard, 2010). Activities and routines are modeled on typical developmental milestones for ages 2 to 24 months reported in the scientific literature ( Chapman, 2000; Gordon-Brannan & Weiss, 2007; MacLeod, 2013; Paul & Norbury, 2012; Stoel-Gammon, 1983; Stoel-Gammon, 2011). Note that there is considerable variability regarding these milestones. An example of such variability is onset of canonical babble among typically developing children ( Oller *et al.*, 1999) as well as children with various types of challenges such as hearing impairment with early amplification ( von Hapsburg & Davis, 2006). Therefore, while the developmental milestones are the same for all children, children in the BBC treatment cohort progress through these milestones not based on their chronological age but on their present levels of ability. Also, activities may be individualized based on a child’s present levels; for instance, one child may need to learn to babble “baba” via shaping the [b] from a raspberry whereas another child might acquire that sound on her/his own or by imitating a model.

Because in young infants, communication skills do not develop in isolation but, rather, in the context of fine and gross motor, cognitive, social-emotional, and adaptive skills, the SLP discusses developmental milestones of the baby as a whole with the parents. An example is a play activity for an 11-month-old child where parent and child take turns putting balls into a bucket. Each time, the parent says, “Ball,” and the child imitates, producing an approximation of the word. This activity builds social-cognitive skills (following directions, imitating, turn-taking), speech and language abilities, and fine and gross motor skills. Learning to greet by waving bye-bye is another example of growth involving social communication and gross motor skill. In the context of the BBC, the SLP makes observations and suggestions regarding those skills most relevant to the communication process, including balance/equilibrium when seated or standing in front of the parent, reaching for objects, and grasping and giving objects.

Following an orientation to the program, the SLP meets with each family once per week for approximately 15 minutes to train and consult on the relevant activities given the child’s current skill status. The SLP obtains information to discuss in each meeting in three ways: (1) Parents send the SLP 1-3 home videos, each up to 2 minutes in length, and the SLP reviews these prior to the meeting; (2) During the meeting, parents report on progress with a given skill that was the focus during that week and on their amount and level of engagement with the child on the skill of interest; and (3) The SLP makes direct observations of the baby and the parent-child interactions during the meeting. In this way, the SLP’s training model includes the following steps: (1) describing the activity/routine, (2) modeling it, (3) giving feedback as parents practice it during the meeting, (4) providing feedback on the home video, (5) evaluating it in the following week’s meeting with the parents by discussing the child’s present skills, and (6) discussing ways to build on these skills towards the next target with the parents. Thus, the SLP follows the teach-model-coach-review approach ( Roberts *et al.*, 2014). Each week, the SLP makes recommendations for no more than two activities/routines to implement that support the child’s current skills and build toward the next developmental step. If parents have questions during the week, they may email the SLP and receive a written explanation. Altogether, the SLP spends 20 to 30 minutes per child per week reviewing the home video, meeting online with the child and parent(s), charting present levels and next goals, and billing.

One key principle underlying all activities is the zone of proximal development or scaffolding in which parents provide speech and language models that bridge what the child can already do and what is slightly beyond the child’s skill set: the model is in the zone of skills that the child can do with help ( Vygotsky, 1979). One key skill targeted throughout the program is imitation.

Fidelity of treatment delivery and implementation is addressed as follows: The same SLP implements the parent training in all families, ensuring consistency and fidelity of this component of the study. All treatment sessions are recorded, and the SLP takes notes on each component of the session (video review, current skills, next steps and recommendations, and discussion of parent questions). She checks to make sure all intervention components of the teach-model-coach-review strategy ( Roberts *et al.*, 2014) are implemented. The SLP provides direct feedback on parent implementation of the activities and routines that were agreed upon in the previous session based on the home videos and live demonstration during the online meetings; this ensures correct implementation of the activities and routines. The SLP further assesses parent fidelity in carrying out the activities and routines by asking the parents how frequently they engaged in the activities/routines during the preceding week. If parents implemented a task or routine with less frequency than agreed upon, the SLP reminds them of the importance of sticking to the plan of action and tells them to contact her right away if they encountered any difficulties. To date, no parent contacted the SLP saying they were unable to implement the activity or routine. Other fidelity measures of parental implementation of the program are percent SLP sessions attended, percent weekly home videos submitted, and percent monthly audio recordings provided. Table 1 summarizes demographic data and fidelity measures of parent follow-through.

### Outcome measures

Because the BBC is designed to beneficially influence speech and language development, the primary outcome variables are speech sound production in babble and speech and expressive language ability. Secondary areas of interests are cognitive and motor development and quality of life, but these are only partially addressed in this pilot and feasibility report. During the active BBC phase, we monitor all of these areas as well as a range of other enrichment variables including volubility of child vocalizations, environmental influences, and demographic factors. Quantitative measures of most of these enrichment variables will be analyzed and described in future reports.

To assess speech sound development and language growth specifically, we use a combination of several standardized tests and established clinical procedures. In the present pilot report, we quantify speech and language growth using several metrics. First, we focus on the complexity of the speech sounds produced during babble and early speech. Once per month per child starting at age 6 months, we compute the Mean Babbling Level (MBL) ( Stoel-Gammon, 1989), a clinical measure of speech sound complexity for babbling. To compute the MBL, a set of at least 50 utterances is compiled and transcribed into the International Phonetic Alphabet using broad transcription. Nonspeechlike vocalizations are excluded from the transcriptions. An expert rater assigns a score of 1, 2, or 3 to each utterance and computes the average; thus, MBL scores range from 1 to 3 for each child at any given time point. A score of 1 is assigned to simple utterances consisting of a vowel, a syllabic consonant, or a consonant-vowel (CV) or vowel-consonant (VC) sequence where the consonant is either a glide (“w”, “y”), a glottal fricative (“h”), or a glottal stop, defined as a brief silence interrupting a vowel. Note that glottal fricatives and stops and glides are not considered to be true consonants. Examples of Level 1 utterances are “m” and “wawa”. A score of 2 is assigned to utterances containing at least one CV or VC sequence with a true consonant; if there are two or more syllables, the consonants may be the same ones or differ only in whether they are voiced or not. Examples are “bapa” and “dida”. A score of 3 is assigned to utterances containing at least two true consonants produced in different parts of the mouth and/or with different airflow characteristics. Examples are “gaba”, and “adap”. The three scores thus express the progression from motorically and linguistically simple to more complex skills. MBL scores in the BBC treatment and control cohort were compared to MBL scores in TD children at equivalent ages as reported in the literature ( Morris, 2010). Whereas the MBL is applied to babble, which does not convey lexical meaning, an equivalent measure called Syllable Structure Level (SSL) exists for meaningful speech ( Paul & Jennings, 1992), and as for the MBL, we used this measure in tandem with typical norms as reported in the literature ( Morris, 2010).

The source materials for the MBL and SSL are daylong naturalistic audio recordings captured with a passive, wearable audio recorder (LENA Research Foundation, Boulder, CO). The recordings are obtained in the natural environment of the families and children. The recorder is returned to the research labs and processed offline to obtain the raw, daylong audio file, which provided the source for the present MBL and SSL scores. Of the other possible measures derived from the recordings, number of child vocalizations per hour is reported here as a potential metric of direct treatment effects and fidelity of parental program implementation, as this metric reflects a behavior that is targeted in treatment. Other metrics derived from the LENA recordings capturing variables known to be important for language and communication development in children will be reported in future publications reporting on larger number of participants. All measures collected in this way are objective and algorithm-driven. For purposes of calculating the MBL and SSL, we extracted multiple 5-minute audio segments with the highest occurrence of child utterances and broadly transcribed these utterances into the International Phonetic Alphabet. These segments were selected using a built-in LENA algorithm, independently of who else is in the acoustic recording environment, for instance one or both parents, siblings, or others. We acknowledge that this sample selection procedure differs from that described in the literature ( Morris, 2010) in that we excerpted segments with the highest volubility of child utterances from day-long recordings, whereas other studies were based on more naturalistic recordings of 30 or 60 minutes’ durations. It is possible that our MBL and SSL scores are higher than they would have been if a more naturalistic 30- or 60- minute sample had been used as the basis for the MBL and SSL scores. In future phases of the project, we will collect data from typically developing children using the same procedures as those described here to create a more equitable basis of comparison.

Second, we used the MacArthur-Bates Communicative Development Inventories 2 (MBCDI-2) ( Fenson *et al.*, 2007) to capture early expressive and receptive vocabulary sizes as reported by the parents who completed the MBCDI-2 protocol forms. MBCDI-2 questionnaires were collected from each family at regular intervals (ages 12, 15, 18, 21, and 24 months), and computed percentile scores were compared between the treated and untreated participants. Percentiles were based on the publisher’s norming sample, separately for boys and girls. The percentiles thus allowed us to compare the study participants to a representative sample of children of equivalent age and gender. Here, we focus on receptive vocabulary, available up to age 15 months, and expressive vocabulary, available for all reported ages.

Third, the Ages and Stages Questionnaires - 3 (ASQ3) parent questionnaires ( Squires & Bricker, 2009) capture communication abilities and personal-social development of young children. The evaluation of communication abilities differs from the MBCDI-2 expressive and receptive vocabulary measures in that it samples a broader range of communication abilities, as relevant for child age, such as comprehending phrases, using language to achieve a goal, and producing multi-word sentences. The personal-social development component queries skills in activities of daily living such as feeding and dressing oneself and social interactions, both verbal and nonverbal, such as giving/receiving objects, asking for help, and role-play, as relevant for age. The ASQ3 is considered valid and reliable for purposes of tracking typical development in these areas and three additional ones (gross motor, fine motor, and problem solving), where the problem solving component is an estimate of cognitive ability ( Schonhaut *et al.*, 2013). All five questionnaire topics are applicable to children with CG because of the known risks for deficits in each of these areas ( Antshel *et al.*, 2004; Karadag *et al.*, 2013; Potter *et al.*, 2013). Each component is scored by summing the raw scores, then assigning one of three categories, based on the provided norms: Above cutoff (“On schedule”), close to cutoff (“Provide learning activities and monitor”), or below cutoff (“Assess further”). The ASQ3 questionnaires are available in 21 separate, age-appropriate and age-normed sets from ages 2 to 60 months. We administer this tool in 6-months intervals and report here on ages 12 and 18 months.

### Data analysis

Results and trends are presented descriptively, supported with graphs. Because of the small sample size of this pilot study, statistical tests for group differences and other inferential statistical procedures cannot be reported here, but will be used in future reports of the study.

### Quality control

Teams of at least two trained research assistants entered questionnaire data from the MBCDI-2 and ASQ3 into the database. Three trained research assistants transcribed the infant utterances and computed MBL and SSL scores. To obtain inter-rater reliability, 10% of the sound files were transcribed again by a different transcriber who also computed MBL and SSL values for the reliability transcription. For these re-transcriptions, samples were selected from all participants representing six different ages ranging from 6 to 22 months. Agreement between the first and second sets of scores was calculated as a percent score where the average difference between the first and second scores, expressed as an absolute value, was subtracted from 100%, as follows:

% Agreement = 100-[(|Score 1 - Score 2|)/(Score 1 + Score 2)/2 × 100] 

The average percent agreement in the reliability transcriptions was 91.5%, which is in line with the reliability agreement of at least 91% reported in the original MBL study ( Stoel-Gammon, 1989) and, hence, consistent with acceptable transcription accuracy. Whereas the participants in the Stoel-Gammon (1989) study were of similar age as the ones in the present study and one of the metrics was identical, the recordings in the Stoel-Gammon study were high-quality analog recordings while we used digital LENA recordings. To our knowledge, there are no comparable MBL studies using LENA recordings. The close similarity in transcriber agreements in the two studies speaks to the acceptable quality of the recordings. The high level of transcriber agreement likely reflects the fact that the transcriptions were broad, not narrow, and that the MBL and SSL scores are not based on segmental identity of phonemes but rather, on phoneme classes ( Morris, 2010). As an additional reliability check, all original MBL and SSL scores were re-calculated by a different team member. Any computational errors detected by a score difference were corrected and the validated values were entered into the final database.

## Results

### Mean Babbling Level (MBL)

**Figure 1.**
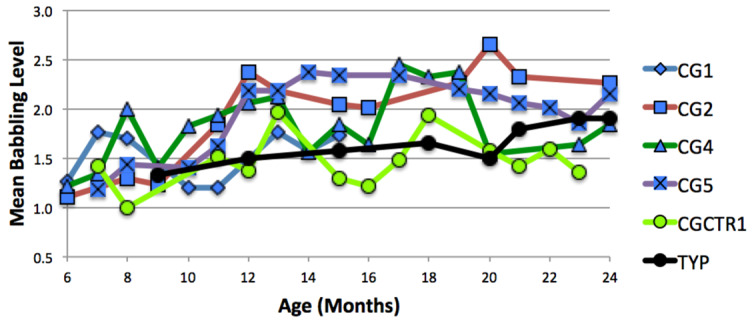
Mean Babbling Level (MBL) scores for the four children with CG in the treatment cohort (blue symbols), the control child with CG (green symbols), and typically developing children (black symbols, archival data from ( Morris, 2010)).

MBL scores of the children with CG in the treatment cohort consistently exceeded those of the control child with CG and typical control children without CG reported in the literature ( Morris, 2010) ( Figure 1). For the ages for which data were available for nearly all five children with CG (7 through 24 months), the average difference between the treatment cohort and the control child with CG was 0.5, indicating that the children with CG in the treatment cohort obtained higher MBL scores than the control child with CG. For the ages for which data were available for the control child with CG and the typical literature-based control children without CG (12, 15, 18, and 20 months, as listed in the literature ( Morris, 2010), the average difference was 0, indicating that the control child with CG obtained MBL scores equivalent to the typically developing children without CG, but note the declining trend for the control child with CG at the most recent ages. As a reminder, the data from the typically developing children reported by Morris (2010) were based on longer, naturalistically obtained recordings, not on 5-minute segments with the highest vocalization rates as in our study. The difference between the children in the treatment cohort and the typical children was -0.34, indicating that the children in the treatment cohort outpaced the typical children on average. Figure 1 shows MBL scores for all children at all available ages.

### Syllable Structure Level (SSL)

**Figure 2.**
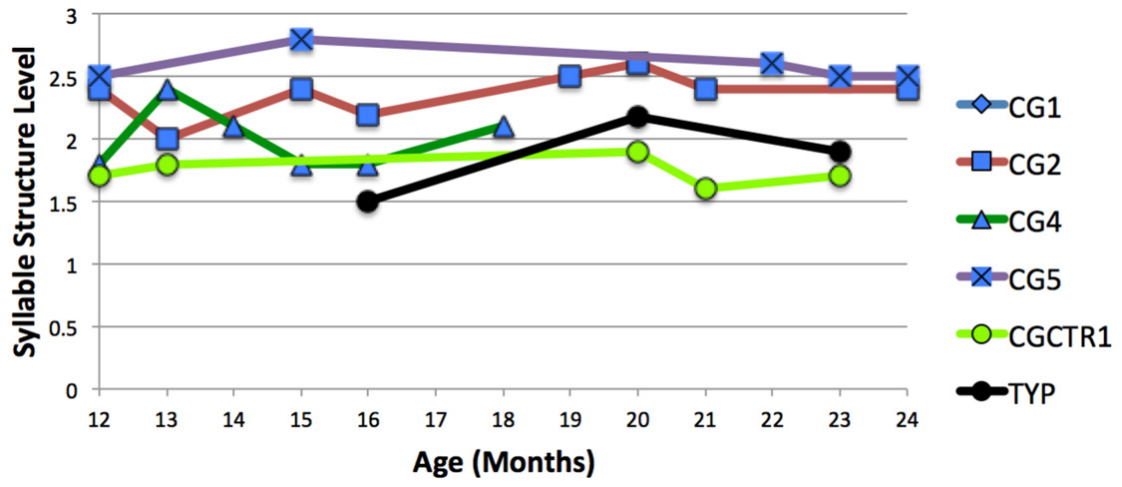
Syllable Structure Level (SSL) scores for the four children with CG in the treatment cohort (blue symbols), the control child with CG (green symbols), and typically developing children (black symbols archival data from ( Morris, 2010)).

The SSL has similar scoring criteria as the MBL, but unlike the MBL, it is based on meaningful speech and required a minimum of 10 utterances. For one child in the treatment cohort, CG1, not enough meaningful utterances could be identified in any of the recording sessions up to 18 months, when the child left the study, to compute the SSL. Of the remaining children and for the available ages, the children in the treatment cohort with CG outperformed the control child with CG by 1.0. For ages 16, 20, and 23 months, the only ages for which data were available for the treatment cohort and the typical children without CG, the children in the treatment group outperformed the typical children by 0.6. For the available datapoints at 20 and 23 months, the control child with CG obtained slightly lower SSL scores than the typical children without CG, by 0.2 points. Note that, with only one exception, the highest scores were obtained by CG2 and CG5. Figure 2 summarizes the SSL scores, where missing datapoints for CG1 and CG4 indicate insufficient numbers of meaningful utterances to calculate the SSL scores

### Expressive and receptive vocabulary

**Figure 3.**
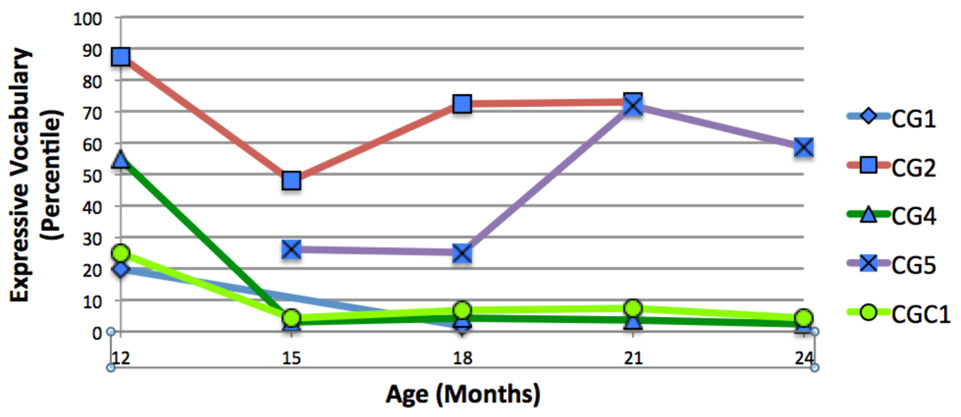
Expressive vocabulary size for ages 12 through 24 months, shown as percentile rankings, for the four children with CG in the treatment cohort (blue symbols) and the control child with CG (green symbols).

Regarding expressive vocabulary size, the control child obtained one of the lowest rankings for the ages for which data were available ( Figure 3). Two children in the treatment cohort, CG2 and CG5, obtained the highest percentile rankings, which, at age 21 months, were 73 and 72, respectively. Two children, CG1 and CG4, obtained low expressive vocabulary scores, with CG1’s percentile score being 1.7 at 18 months and CG4’s being, 4.0 at 21 months. The control child, similar to CG1 and CG4 in the treatment cohort, showed declining expressive vocabulary scores, below the 8 ^th^ percentile at 21 months. Note that not having any words at all at age 12 months, which was the case for CG1 and the control child, corresponds to the 25 ^th^ percentile for boys and the 20 ^th^ percentile for girls, but the ranking drops rapidly with age if the expressive vocabulary does not increase substantially. Figure 3 summarizes the expressive vocabulary percentiles.

**Figure 4.**
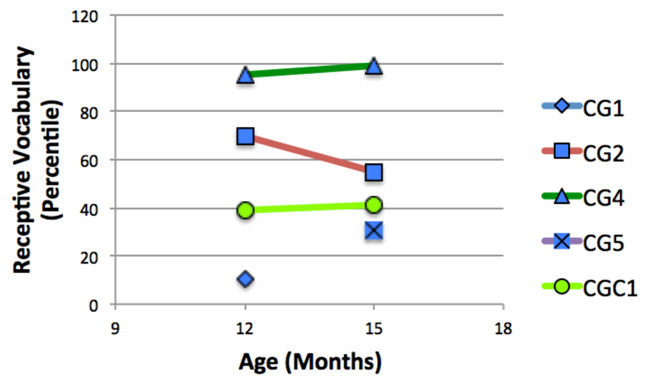
Receptive vocabulary size for ages 12 and 15 months, shown as percentile rankings, for the four children with CG in the treatment cohort (blue symbols) and the control child with CG (green symbols).

Regarding receptive vocabulary, the control child obtained the second-lowest percentile ranking ( Figure 4), but note that all children including the control child show typical receptive vocabulary scores at the two ages represented in the questionnaires. The lowest percentile score, 10.5, is considered low average; it was obtained by CG1 at age 12 months. All other scores are solidly within normal limits. Figure 4 summarizes receptive vocabulary percentiles, respectively.

### Automatized metrics

**Figure 5.**
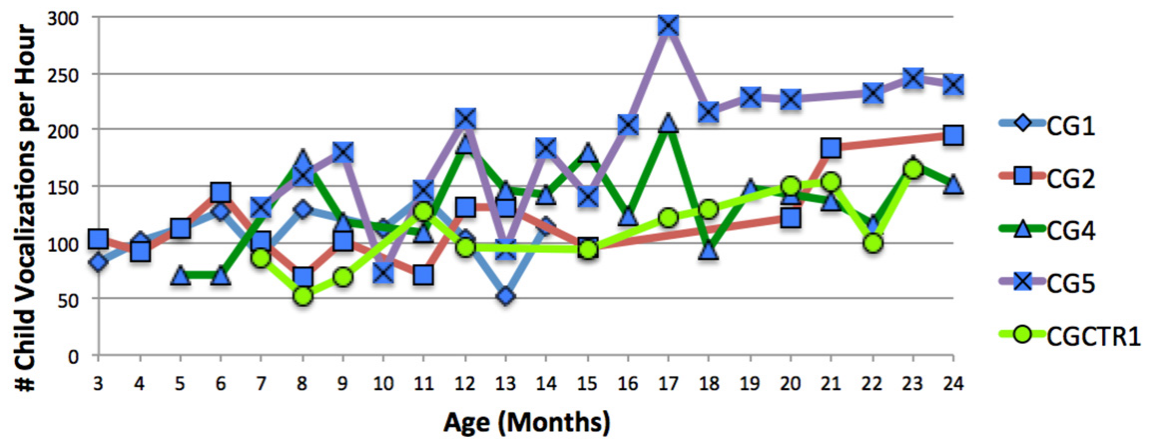
Number of child vocalizations per hour for ages 3 through 24 months for the four children with CG in the treatment cohort (blue symbols) and the control child with CG (green symbols).

All children except CG1 showed growth over time in their vocalization rates per hour. Figure 5 shows highest vocalization rates for CG2 and CG5 and low vocalization rates for CG4 and the control child with CG, especially towards age 24 months. For those ages for which data were available for at least two of the children in the treatment cohort and the control child with CG, the children in the treatment cohort averaged 46 vocalizations per hour more than the control child with CG.

### Ages and Stages Questionnaires -3 (ASQ3)

The AGS3 questionnaires at 12 and 18 months indicated that three of the four children in the treatment cohort had communication and personal-social abilities as expected for age. The communication performance of one child in this cohort was below cutoff for age at 18 months, and the personal-social performance was close to the cutoff for age expectations. The control child scored close to the cutoff for age expectation at 18 months for communication, and at 12 and 18 months for personal-social abilities.

Regarding problem solving performance, of the children in the treatment cohort, CG1 and CG4 had scores that were not consistently in the range of scores expected for age, both at age 18 months, whereas the control child with CG was close to the cutoff at both time points. Regarding fine and gross motor skills, CG1 was the only child in the treatment cohort with scores that were below (fine motor) or close to (gross motor) the cutoff, whereas the control child had scores close to the cutoff in both areas at one time point and, for gross motor, at both time points. Across all areas, mainly one child in the treatment cohort (CG1) and the control child did not show developmental skills on schedule. Table 2 summarizes the ASQ3 scores for all children in the five assessed areas.

**Table 2.** Ages and Stages Questionnaires- 3 (ASQ3) scores for ages 12 and 18 months.

Domain	CG1		CG2		CG4		CG5		CTR1	
	12 M	18 M	12 M	18 M	12 M	18 M	12 M	18 M	12 M	18 M
Communication	AC	BC	AC	AC	AC	AC	AC	AC	AC	CC
Personal-Social	CC	AC	AC	AC	AC	AC	AC	AC	CC	CC
Problem Solving	AC	CC	AC	AC	AC	BC	AC	AC	CC	AC
Fine Motor	AC	BC	AC	AC	AC	AC	AC	AC	AC	CC
Gross Motor	CC	AC	AC	AC	AC	AC	AC	AC	CC	CC

*Note:* AC = Above cutoff (on schedule), BC = Below cutoff (assess further), CC = Close to cutoff (monitor)

## Discussion

In a small sample of very young children with a known and highly predictive risk for speech and language disorders due to CG, we provide preliminary evidence that a program of proactive activities and routines, the BBC, may have beneficial effects in several important regards.

First, very early attention to vocalizations and speech sound production may increase children’s ability to produce more complex speech sounds in babble and speech. The children with CG in the treatment cohort obtained greater MBL scores during babble than the control child with CG as well as typical control children without CG. A similar pattern was seen for the SSL during meaningful speech. Note that the MBL and SSL scores in our study were calculated based on 5-minute segments with the highest volubility, whereas the typical MBL and SSL scores reported in the literature were based on longer samples regardless of volubility. Therefore, the higher scores in the treatment cohort, compared to typically developing children described in the literature, may well be an artifact of this difference in methods. The differences between these metrics in the treatment cohort, compared to the control child with CG, however, are meaningful because the same sample selection was applied to all of these participants. Together, the MBL and SSL findings suggest that children with CG may benefit from very early attention to vocal and speech sound production in terms of increased complexity in babble and speech. It is possible that MBL and SSL are adequate measures for areas of crucial weakness in children with CG in general, and that targeting speech sound skills may give them a clear boost that may have beneficial effects on later speech and language development. Our fully powered clinical trial, launched two years after the pilot phase began and currently in progress, is likely to provide conclusive evidence in this regard. This larger clinical trial includes three cohorts, one with children who have CG and receive both the BBC treatment and the close monitoring via questionnaires, monthly day-long audio recordings, and standardized testing, one with children who have CG and receive the close monitoring only, and one with typical controls who also only receive the close monitoring.

Second, the expressive vocabulary, and possibly to a lesser degree the receptive vocabulary, of children with CG may improve as a result of the BBC. Expressive vocabulary scores as measured with the MBCDI-2 were completely within normal limits for two children in the treatment group, CG2 and CG5, whereas the control child with CG and two children in the treatment cohort, CG1 and CG4, scored below expectation. Given that over half to two thirds of children with CG exhibit language impairment, low language skills in the control child would be expected. Finding two of four children in the treatment cohort with typical language skills may or may not show major beneficial effects of the BBC program; more extensive language measures at subsequent ages will provide a more complete picture. The fact that receptive vocabulary scores were within normal limits for all children including the control child with CG may indicate that CG affects expressive language skills more than receptive language skills.

Third, there may be gains in competence in using language and nonverbal means to interact with others. Three of the children in the treatment cohort had communication skills and personal-social skills as expected for age, as measured with the ASQ3. These children may be gaining age-appropriate competence in activities of daily living as well.

Comparing the results from all measures, the two children with the overall highest scores were CG2 and CG5. They showed no evidence of deficits in any of the evaluated areas; in fact, in the areas of speech sound complexity in meaningful speech and expressive vocabulary size, they scored at the top of the pilot sample. By contrast, CG1 did not produce enough meaningful speech for SSL scores, had low expressive vocabulary scores, and scored near or below the cutoff for expectation in all five developmental domains assessed with the ASQ3. CG4 had intermediate MBL and SSL scores but low expressive vocabulary scores and one low score in the AGQ3, namely in problem solving. The control child with CG obtained some of the lowest MBL, SSL, expressive vocabulary, and ASQ3 scores. These patterns are consistent with cross-domain associations among the evaluated skills (speech, language, social interaction, cognitive ability, motor ability). Whether these patterns reflect global levels of disease severity, global benefits from all of the components of the treatment, or a combination of these cannot be ascertained based on the present data.

Some evidence for the efficacy of the treatment is found in the child vocalization rates per hour. Generally, vocalization rates were higher in the treatment cohort, compared to the control child with CG, and they patterned with outcome measures of speech sound complexity and expressive vocabulary. This growth in child vocalization rates in the treatment cohort may be a result of parental fidelity in carrying out the activities and routines, and it may also represent a direct effect on child behaviors, as increases in child vocalization rates are targeted in the treatment activities and routines. Of the other measures that potentially reflect fidelity of parental follow-through, percent attendance of weekly SLP sessions seemed to predict outcomes best, although CG4 attended nearly all sessions but patterned with the control child across outcome measures. Whether an induced growth in vocalization rates is just one of many effects of the treatment, or whether it has a facilitatory contribution on more advanced speech and language outcomes remains to be explored once a larger dataset is available. 

### Limitations and future directions

The goals of this study were to obtain preliminary results and to document feasibility of the methods. Both of these goals were accomplished. Because of the small sample size in this pilot study, generalizations to other children with CG are not possible. Whereas the MBCDI-2 percentiles were based on sex-adjusted norms, the other measures were not, but note that the two children with the overall highest performance were a girl and a boy. It is not possible to identify which components of the BBC had the greatest impact, if any, on speech, language, cognitive, and motor development. Many other variables in the study, for instance quantity of child-directed speech, remain to be analyzed, not only in this pilot cohort but also in the full set of families in this study. Longer-term outcomes in the primary and secondary outcome variables will be evaluated as we follow the children until age 4 years and investigate a more complete spectrum of outcome variables including speech, language, cognitive and motor development, and quality of life. Most importantly, the trends toward beneficial effects of the BBC on the primary outcome variables of speech sound production and expressive language warrant appropriately powered larger clinical trials.

Currently, a fully powered clinical trial is in progress (R01 HD098253-02). For this larger study, we have created a process-based fidelity check for parents to ensure that they implement the activities and routines as intended, and we continue to use the process-based fidelity checks to ensure that the SLP regularly completes all components of the intervention. We are making efforts to minimize barriers to implementation fidelity by closely monitoring for factors that can diminish implementation fidelity ( Cane *et al.*, 2012; Justice *et al.*, 2015). Results will be published following standardized reporting procedures using the CONSORT checklists and templates ( Hoffmann *et al.*, 2014; Ludemann *et al.*, 2017). As the project expands in terms of research purview and additional SLPs are onboarded, and also as the Babble Boot Camp approaches widespread use among clinicians, a detailed instructional project manual and a videorecorded tutorial are planned, along with coaching and fidelity checks to safeguard that SLPs implement the program accurately and consistently.

## Data availability

### Underlying data

Open Science Framework: Babble Boot Camp. https://doi.org/10.17605/OSF.IO/YZHT4 ( Peter, 2019)

The project contains the following underlying data files:

- UnderlyingData_MBL_SSL_MBCDI2.xlsx. This spreadsheet contains the MBL, SSL, and MBCDI-2 data for the participants.

The underlying data also include audio files recorded in the participants’ homes and hand-written questionnaires with identifiable information; therefore, these cannot be openly shared. De-identified questionnaire files can be made available to qualified researchers by contacting the first author at Beate.Peter@asu.edu.

### Extended data

Open Science Framework: Babble Boot Camp. https://doi.org/10.17605/OSF.IO/YZHT4 ( Peter, 2019)

The project contains the following extended data files:

- Extended Data 1: Flow diagram
- Extended Data 2_Appendix_BBC_Program_v2

### Reporting guidelines

Open Science Framework: Babble Boot Camp. https://doi.org/10.17605/OSF.IO/YZHT4 ( Peter, 2019)

The project contains the following reporting guidelines:

- Reporting Guidelines 1: CONSORT checklist with Information to include in the abstract when reporting a pilot or feasibility trial
- Reporting Guidelines 2: CONSORT checklist with Information to include in the manuscript when reporting a pilot or feasibility trial

Data are available under the terms of the Creative Commons Attribution 4.0 International license (CC-BY 4.0).
