# Supplementary material for: Toward a paradigm shift from deficit-based to proactive speech and language treatment: Randomized pilot trial of the Babble Boot Camp in infants with classic galactosemia
Source: F1000Res. 2020 Jul 6;8:271. Originally published 2019 Mar 11. [Version 5] doi: 10.12688/f1000research.18062.5 (PMC7291108; doi:10.12688/f1000research.18062.5)
Supplement: Peer review history

# Peer review: Reviewer response for version 5

**DOI:** 10.5256/f1000research.27718.r66483

**Published:** 2020-07-27

**Authors:** Rebecca McCauley

**Affiliations:** 1 Department of Speech and Hearing Science, The Ohio State University, Columbus, OH, USA

I appreciate the authors' careful attention to my previous comments and look forward to hearing from this group as their work continues.  Pushing early intervention into the arena of prevention makes such good sense!

Is the work clearly and accurately presented and does it cite the current literature?

Yes

If applicable, is the statistical analysis and its interpretation appropriate?

Yes

Are all the source data underlying the results available to ensure full reproducibility?

Yes

Is the study design appropriate and is the work technically sound?

Yes

Are the conclusions drawn adequately supported by the results?

Yes

Are sufficient details of methods and analysis provided to allow replication by others?

Partly

Reviewer Expertise:

Developmental speech sound disorders, methods related to the description of behavioral interventions in developmental communication disorders.

I confirm that I have read this submission and believe that I have an appropriate level of expertise to confirm that it is of an acceptable scientific standard.

# Peer review: Reviewer response for version 4

**DOI:** 10.5256/f1000research.25295.r63031

**Published:** 2020-06-11

**Authors:** Rebecca McCauley

**Affiliations:** 1 Department of Speech and Hearing Science, The Ohio State University, Columbus, OH, USA

Version 4 is the first version of this report I have had an opportunity to review. The two major concerns I have are the need for more information concerning (1) the treatment procedures executed by parents and the speech-language pathologist (SLP) and (2) the fidelity with which they apply those procedures during child-parent interaction and parent training, respectively. I also will address a less substantial concern that was raised in a previous review. My recommendations on these issues include some steps that I believe should be taken as part of an additional revision as well as others that should be incorporated in the larger RCT.

**Treatment Procedure Description. **More detailed information about treatment procedures is needed in part because of the complex way in which the intervention is delivered: indirectly, through parent training and ongoing coaching by the speech-language pathologist and directly, through interactions with the child by the parents. Currently, in their description of treatment procedures undertaken by parents, the authors provide explicit descriptions for only one activity and one routine, although 17 activities and routines comprise the bulk of the procedures they will enact. In addition, in the description of training, coaching and procedures undertaken by the SLP, it is hard to tell which procedures are considered key to achieving outcomes. 

 To address these issues in the current report, I recommend that the authors provide readers with access to the intervention protocol or manual specifying each activity and routine being taught to the parents and further specification of the procedures incorporated by the SLP as part of parent training and coaching. Additionally, for the larger RCT, I recommend that the authors consult and make use of not only the CONSORT checklist related to pilot and feasibility trials, but also TIDieR: Template for Intervention Description and Replication (Hoffman *et al*., 2014) ^1^. Recent concerns about the transparency and replicability of interventions along with subsequent developments in the use of reporting guidelines suggest the importance, for both research and applied purposes, of the thorough description of interventions (Ludemann *et al*., 2016) ^2^, particularly those that are complex in nature.

**Fidelity**. With regard to future fidelity procedures, I join the first reviewer of this version of the manuscript in recommending the addition of a process-based approach to the measurement of fidelity for treatment procedures undertaken by parents. In addition, I would recommend a similar approach be adopted for addressing the fidelity of the SLP training and coaching activities. In the current version of the study report, readers are assured that “The same SLP implements the parent training in all families, ensuring consistency and fidelity of this component of the intervention” (p. 6). Since one might expect more SLPs to become involved in parent training as the number of participants increases over the course of the RCT, the need to evaluate fidelity is likely to become substantially more pressing. Still, even were a single SLP able to provide training for all families, it would be valuable to verify that training methods do not vary substantially across children not because of *appropriate* attention to addressing each child’s specific needs but because the clinician’s attention, memory or other resources important to fidelity are taxed by factors that may be related to characteristics of the child, the parent, or other factors (Cane *et al*., 2012; Justice *et al*., 2015) ^3^^,^^4^. Further, because a process-based approach will focus on possible key ingredients of the training, increased attention to this aspect of the methods may also promote the development of valuable supports to fidelity (e.g., checklists) with eventual benefits to Babble Boot Camp’s widespread adoption and use with fidelity by non-research SLPs.

**Response to earlier concerns regarding IPA transcriptions. **A somewhat less substantive concern relates to the reporting of agreement data for transcriptions in this version of the study report. An earlier review raised the concern that APA transcription from LENA recordings was not supported by the manufacturers of that device and could seriously undermine the reliability/agreement of the outcome measures based on them. In this version of the manuscript, additional information about agreement methods is provided and the average percentage difference in pairs of transcriptions is reported as 8.5%. This finding was described as “consistent with acceptable transcription accuracy” (p. 5). I’d like to suggest that a citation is needed to support that characterization, ideally one based on transcriptions performed under similar conditions those reported here (i.e., with data obtained using Lena) as well as under more typical transcription conditions. Finally, I believe that it would be more appropriate to use the term “agreement” rather than either “reliability” or “accuracy” in discussion of this subject (Stolarova et al., 2014) ^5^.

 These comments are offered in hopes of supporting the ongoing success of this ambitious and important line of inquiry.

Is the work clearly and accurately presented and does it cite the current literature?

Yes

If applicable, is the statistical analysis and its interpretation appropriate?

Yes

Are all the source data underlying the results available to ensure full reproducibility?

Yes

Is the study design appropriate and is the work technically sound?

Yes

Are the conclusions drawn adequately supported by the results?

Yes

Are sufficient details of methods and analysis provided to allow replication by others?

Partly

Reviewer Expertise:

Developmental speech sound disorders, methods related to the description of behavioral interventions in developmental communication disorders.

I confirm that I have read this submission and believe that I have an appropriate level of expertise to confirm that it is of an acceptable scientific standard, however I have significant reservations, as outlined above.

## References

[1] : Better reporting of interventions: template for intervention description and replication (TIDieR) checklist and guide. *BMJ*.2014;348: 10.1136/bmj.g1687 g1687 10.1136/bmj.g1687 24609605

[2] : Investigating the Adequacy of Intervention Descriptions in Recent Speech-Language Pathology Literature: Is Evidence From Randomized Trials Useable?. *Am J Speech Lang Pathol*.2017;26(2) : 10.1044/2016_AJSLP-16-0035 443-455 10.1044/2016_AJSLP-16-0035 28475801

[3] : Validation of the theoretical domains framework for use in behaviour change and implementation research. *Implement Sci*.2012;7: 10.1186/1748-5908-7-37 37 10.1186/1748-5908-7-37 22530986PMC3483008

[4] : Designing Caregiver-Implemented Shared-Reading Interventions to Overcome Implementation Barriers. *J Speech Lang Hear Res*.2015;58(6) : 10.1044/2015_JSLHR-L-14-0344 S1851-63 10.1044/2015_JSLHR-L-14-0344 26262941PMC4816633

[5] : How to assess and compare inter-rater reliability, agreement and correlation of ratings: an exemplary analysis of mother-father and parent-teacher expressive vocabulary rating pairs. *Front Psychol*.2014;5: 10.3389/fpsyg.2014.00509 509 10.3389/fpsyg.2014.00509 24994985PMC4063345

# Response

**Published:** 2020-06-27

**Authors:** Beate Peter

**Affiliations:** Arizona State University, USA

Many thanks for this very thorough and helpful review. We greatly appreciate the suggestions for the present manuscript and also for the larger clinical trial regarding barriers to implementation fidelity and reporting standards. We have addressed the concerns as follows:

- Added an appendix that lists and describes all intervention components of the study. The descriptions of the activities and routines were excerpted from the program overview that parents receive at the start of the intervention.

- Contextualized our transcription reliability by converting the accuracy measure from differences to agreement and by showing that our agreement rates were nearly identical to those in the original MBL paper.

- Outlined the implementation and reporting procedures in place for the clinical trial that followed the pilot study described in the present manuscript.

- Described implementation and fidelity measures planned for future expansions of this project, not only in terms of additional research but, eventually, in terms of clinical practice.

We made a few additional minor changes, e.g., corrected some typographical errors that had previously escaped notice and updated some of the information.

# Peer review: Reviewer response for version 4

**DOI:** 10.5256/f1000research.25295.r61857

**Published:** 2020-04-14

**Authors:** Barbara L. Davis

**Affiliations:** 1 Department of Communication Sciences and Disorders, University of Texas at Austin, Austin, TX, USA

I reviewed the Version 4 revision for the Peter *et al*. submission following review of versions 1 and 2. The present narrative is dramatically improved overall in the detail given on child outcomes and, generally, on limitations and caveats of the present pilot study.

 My major concerns in my earlier reviews were first, that there were no measures of parent training fidelity and second, the status of the typically developing cohort was imprecise.

 Relative to the issue of parent training, the author’s rationale for their present focus on measuring child variables was much stronger. However, because this large study is poised to be of great value to researchers and clinicians focused on the earliest stages of pre-linguistic development in high risk populations, I would like to point out an issue that could make this study a stronger contributor to the parent training literature. In the *Materials and Procedures* section (final paragraph), the authors expand their description of “parent fidelity in carrying out routines” by detailing a number of legitimate frequency- based metrics of evaluation of parent behaviors (i.e., “how frequently they engaged in the activities/routines during the preceding week”, ..…."percent SLP sessions attended, percent weekly home videos submitted, and percent monthly audio recordings provided”). These process-based metrics are excellent, but I feel that the authors, in the main body of their study could push further into the content-based quantification of the parent-child interactions to collect data on the actual interactions themselves between the parents and their children, not only the frequency metrics (noted above). These kinds of content-based quantifications could be invaluable to researchers and clinicians in this area. By content, I am indicating counting dyadically-based behaviors such as the following:

1. increase in maturity of vocalizations following modeling by the parent; see Stoel-Gammon or Oller measures of vocalization maturity;
2. parental use of parallel talk or expansions;
3. dyadic balance of turn taking;
4. dyadic balance in maintenance of eye contact.

These content-based behaviors are intended as examples, not an exhaustive list, but all are based on available research into earliest periods of typical infant development of pre-linguistic vocal capacities. They enable the researcher to specify how the parents accomplished their dyadic interactions not only the frequency of compliance with study design requirements. In short, I am urging the research team to expand their quantification from frequency-based metrics into content-based metrics, potentially to identify more closely potential aspects of parent behaviors that might map onto successful or less successful outcomes in their larger child cohort followed in the larger study.

 Relative to the issue of the typically developing child cohort, the authors did an adequate job of outlining caveats to their comparison cohort relative to the length (i.e. 5 minutes versus 30-60 minutes) and types of data (i.e. highest volubility samples versus functional use samples) selected for typical development comparison samples. I would like to see an ongoing attention to this issue in the larger study.

 Overall, the revisions completed are adequate to index the pilot data for an ongoing larger research study. My present comments are intended to support use of this pilot data to consider expansions into parent-child dyadic interactions in this critical formative period.

Is the work clearly and accurately presented and does it cite the current literature?

Partly

If applicable, is the statistical analysis and its interpretation appropriate?

No

Are all the source data underlying the results available to ensure full reproducibility?

No

Is the study design appropriate and is the work technically sound?

Partly

Are the conclusions drawn adequately supported by the results?

No

Are sufficient details of methods and analysis provided to allow replication by others?

Partly

Reviewer Expertise:

Early periods of child speech acquisition in typically developing and at-risk populations.

I confirm that I have read this submission and believe that I have an appropriate level of expertise to confirm that it is of an acceptable scientific standard.

# Peer review: Reviewer response for version 3

**DOI:** 10.5256/f1000research.24580.r59625

**Published:** 2020-03-02

**Authors:** Shelley Velleman, Claudia Abbiati

**Affiliations:** 1 Interdisciplinary PhD Program in Human Functioning and Rehabilitation Science, University of Vermont, Burlington, VT, USA; 2 Department of Communication Sciences and Disorders, University of Vermont, Burlington, VT, USA

Two major limitations remain in this manuscript:

 1. The authors still have not explained - or perhaps have not carried out – the agreement procedures for the study. In their responses to the previous reviews, they merely stated that, “We reanalyzed the entire dataset in terms of MBL and SSL and found very little discrepancies between the first and second dataset.” This is insufficient. The speech samples were gathered via LENA, which is not appropriate for IPA transcription (as admitted freely by the LENA developers themselves). Yet, the 5-minute samples were transcribed in broad IPA for analysis in this study. No information is provided about inter-rater or intra-rater agreement among transcribers, which is likely to be unacceptably low for LENA samples.

 Given that these transcripts were used for MBL and SSL analysis, it is actually not crucial that the transcribers agreed on the specific consonants and vowels that were produced in the samples. BUT for these MBL and SSL analyses to have any validity, the transcribers must have good agreement upon:

 a.     syllable shapes – inclusion of true consonants (vital for differentiating level 1 vs. levels 2 or 3)

 b.     word shapes – i.e., whether a given vocalization contains one or more syllables (vital for differentiating level 2 vs. level 3)

 c.     consonant differentiation – whether or not the consonants in vocalizations of 2 syllables or more were harmonized i.e., whether the syllables within each di/multisyllabic vocalization were reduplicated (vital for differentiating level 2 vs. level 3).

 Therefore, an appropriate reliability procedure would be to calculate the MBL or SSL for a selected percentage of children at a sample of ages based on two different transcriptions, to see if the same values would result from the two transcripts. The results of this procedure should be reported *in the article*. 

 2. The samples used in this study were “multiple 5-minute audio segments with the *highest occurrence* of child utterances” (p. 7). On the other hand, the samples reported in the literature to which the treatment children are compared were *not* selected to be samples during which the typical children were especially voluble. They were 30-minute (or sometimes 60-minute) samples that were used as is, regardless of the child’s volubility at the time. Therefore, it is *not* appropriate for the authors to claim that the children in the present study performed better on these measures than the children reported in previous literature (e.g., on p. 7: “the children in the treatment cohort outpaced the typical children”). It is a comparison of the best moments of the treatment children versus typical moments of the children from the literature. We cannot know what the MBL and SSL levels of the typical children would have been if 5-minute selections of their most voluble moments of a day had been selected out for analysis. 

 There are still a few typos, etc. in the manuscript even though the authors did correct most of the ones that we (not Reviewer 2) pointed out before.

Is the work clearly and accurately presented and does it cite the current literature?

Partly

If applicable, is the statistical analysis and its interpretation appropriate?

Not applicable

Are all the source data underlying the results available to ensure full reproducibility?

No

Is the study design appropriate and is the work technically sound?

Partly

Are the conclusions drawn adequately supported by the results?

Partly

Are sufficient details of methods and analysis provided to allow replication by others?

No

Reviewer Expertise:

Infant, toddler, and child speech development and disorders, including prelinguistic vocalizations and early words, and motor speech development and disorders.

We confirm that we have read this submission and believe that we have an appropriate level of expertise to confirm that it is of an acceptable scientific standard, however we have significant reservations, as outlined above.

# Response

**Published:** 2020-03-17

**Authors:** Beate Peter

**Affiliations:** Arizona State University, USA

We appreciate Reviewer 1’s third review. In the previous revision, we had addressed Reviewer 2’s concerns and addressed Reviewer 1’s new concerns as follows:

Inter-rater reliability: To obtain inter-rater reliability, 10% of the sound files were transcribed by a different transcriber who also computed MBL and SSL values for the reliability transcription. Differences between the first and second scores were calculated as an absolute value and expressed as a percent score relative to the average difference between the first and second scores. The average difference amounted to 8.5%, which is acceptable. The second reliability procedure, a complete recalculation of all MBL and SSL scores by a different team member, was based on the original transcriptions. Any computational errors that were identified with a discrepancy between the first and second score were corrected and the validated values were entered into the final database.

MBL and SSL sample selection: Reviewer 1 pointed out that studies of typical children are based on naturalistic half- or whole-hour recordings whereas we selected 5-minute segments with the highest volubility from a day-long recording. This is an excellent point regarding our study design that ideally would have been made in the first round of review. We added explanatory text to the Methods and revised the interpretation of our results, which make a lot more sense now.

Writing style: We had implemented 11 of the 16 suggestions for improving the writing style. Of five cases flagged as run-on or long sentences, we have now broken down two into shorter components but in the remaining three, we preferred our own wording. In this revision, we made a few additional improvements of spelling and punctuation. Since no details regarding style were mentioned in this review, we trust that our writing style is acceptable now.

# Peer review: Reviewer response for version 2

**DOI:** 10.5256/f1000research.22449.r53504

**Published:** 2019-10-10

**Authors:** Shelley Velleman, Claudia Abbiati

**Affiliations:** 1 Interdisciplinary PhD Program in Human Functioning and Rehabilitation Science, University of Vermont, Burlington, VT, USA; 2 Department of Communication Sciences and Disorders, University of Vermont, Burlington, VT, USA

This article is much improved. The literature review is far more comprehensive and the Methods section has also been expanded. The reader is now able to appreciate and evaluate the research more thoroughly and objectively.

 The primary remaining weaknesses still lie in some of the descriptions of the methods used. Of most concern is the assessment of parent fidelity. The authors have now disclosed that “The SLP assesses parent fidelity in carrying out the activities/routines that had been agreed upon in the previous session, by asking the parents *how frequently they engaged in the activities/ routines* during the preceding week.” (p. 5; italics added). However, assessment of the quality of the parent treatment does not appear to be measured. It is possible that this is done through the review of the “..up to three home videos up to 2 minutes in length” that the parents submit (weekly?). However, the content of these videos is not currently described in the article. It is also important to know how many videos, of what lengths, were actually received for the participants described in this report and what they revealed about these specific parents’ fidelity to the content/quality of the treatment protocol.

 Another aspect of the methods that should be clarified relates to the MBL and SSL calculations. The authors report that 15% of the infants’ “scores” were “double-scored” by other team members (p. 7) with differences over 10% resolved by consensus (if needed). However, it’s not clear what this means. Were the transcripts re-transcribed, recoded for MBL and SSL, and the scores (means) then recalculated? Or was the “double-scoring” only recoding of each vocalization with respect to MBL or SSL and then recalculating the means of each? If only the calculation of the means was redone (no re-transcribing or recoding), that is not sufficient. At least the MBL and SSL coding should be redone for reliability’s sake.

 The MBL and SSL comparisons to TD children should also be explained in more detail. For example, how many TD children were in the comparison samples? What were the characteristics of those children? In addition, the MBL and SSL scores for the participants in this study were based on “multiple 5-minute audio segments *with the highest occurrence of child utterances*” (p. 6; italics added). Was this selection process of segments with many utterances also used in the TD comparison samples? If not, this difference could at least partially account for the apparently superior performance of some of the children with CG in comparison to the TD samples. In addition, it is stated earlier on the same page that “a set of at least 50 utterances is compiled” for each participant. Do the authors really mean 50 utterances or do they mean 50 vocalizations? It would be helpful to specify how many minutes were required for each of the participants reported in this study to amass 50 utterances and how many vocalizations they produced per utterance.

 We find it mysterious that [d] could be shaped from a raspberry (page 5, column 1, 2 ^nd^ paragraph in Materials and Procedures, last sentence). Please correct or clarify.

 The figures are much easier to interpret now, but we wonder if boxplots (comparing treated CG participants vs. control participant vs. TDs) might be more revelatory (either instead of the current figures or as well as the current figures), since they would show the distributions of scores better.

 Other questions also remain to be answered. For example, it would be helpful if the review of the literature included information about the ages at which deficits in speech, language, fine motor, and gross motor skills typically appear in children with CG. Are the children in this study younger than the ages of emergence of those deficits or are the problems typically identified very early on?

 We have reviewed the article in detail and have quite a few specific suggestions for improving the writing style and clarity. These are listed below. Many of these relate to long, wordy, complex sentences that would be much more reader-friendly if they were shortened, re-organized, or broken into multiple sentences. Only the most egregious cases are listed here; the authors are encouraged to read through the ms. to identify other sentences that could be clarified in these ways. Only the first several words of the sentence are provided in the list below to indicate sentences that should be simplified.

- Pg. 3, Column 1, paragraph 1, line 9 – run-on sentence: “There is strong evidence that …”.
- Pg. 3, column 2, 1 ^st^ full paragraph, 13 lines up from bottom: “… no study included children younger than 12 months or targeted prelinguistic skills.” This should read, “younger than 12 months ***and*** targeted prelinguistic skills.” There have certainly been studies, particularly in ASD, that have addressed prelinguistic skills beyond 12 months of age.
- Pg. 3, column 2, 1 ^st^ full paragraph, 8 lines up from end of paragraph – long sentence: “Going even further back in the developmental trajectory, …”.
- Pg. 3, column 2, last paragraph, 8 lines up: long sentence - “Despite rigorous dietary management…”.
- Pg. 3-4, last sentence on p. 3 going over to p. 4: long sentence - “Speech disorders were reported…”.
- Pg. 4, column 1, line 9: State clearly what is the current “conventional management”?
- Pg. 4, column 1, 1 ^st^ full paragraph, 1 ^st^ sentence: Replace “children” with “infants.” (“…designed for children…”). Same for other references of participants as “children” throughout the ms.
- Pg. 4, column 1, 1 ^st^ full paragraph, last sentence: Insert “other”: “…then progresses through all other prelinguistic and…” (because eye contact and pre-babble vocalizations are also prelinguistic stages).
- Pg. 4, Last paragraph, 4 lines up: Awkwardly worded - “In all five families…”.
- Pg. 5, 1 ^st^ sentence of Materials and Procedures: Insert the word “intervention”, or something like that, after “with expertise in early childhood ______.”
- Pg. 5, Materials and procedures paragraph 2, 14 lines up: long sentence - “Note that there is considerable…”.
- Pg. 5, column 1, last paragraph, 1 ^st^ sentence: long sentence - “Because in young infants…”.
- Pg. 6, column 1, 1 ^st^ full paragraph, sentence 1: Move “Specifically”: “To assess speech sound development and language growth specifically, we use…”
- Pg. 6, column 1, 1 ^st^ full paragraph, 15 lines down: long sentence - “A score of 1 is…”
- Pg. 9, column 2, line 4: Awkwardly worded - “Our fully powered clinical trial, recently…” Also, insert “more”: “…is likely to provide more conclusive evidence…”
- Pg. 9, column 2, 2 ^nd^ paragraph of Discussion, line 1: Change “speech sound production” to “vocal production” since this includes pre-babble?

Is the work clearly and accurately presented and does it cite the current literature?

Partly

If applicable, is the statistical analysis and its interpretation appropriate?

Not applicable

Are all the source data underlying the results available to ensure full reproducibility?

No

Is the study design appropriate and is the work technically sound?

Partly

Are the conclusions drawn adequately supported by the results?

Partly

Are sufficient details of methods and analysis provided to allow replication by others?

No

Reviewer Expertise:

Infant, toddler, and child speech development and disorders, including prelinguistic vocalizations and early words, and motor speech development and disorders.

We confirm that we have read this submission and believe that we have an appropriate level of expertise to confirm that it is of an acceptable scientific standard, however we have significant reservations, as outlined above.

# Peer review: Reviewer response for version 2

**DOI:** 10.5256/f1000research.22449.r53505

**Published:** 2019-09-17

**Authors:** Barbara L. Davis

**Affiliations:** 1 Department of Communication Sciences and Disorders, University of Texas at Austin, Austin, TX, USA

*Introduction*

 The Introduction section is much improved and the focus on child outcomes is made clear. The rationale for the study is also well done in this section of the manuscript. However, the authors review literature on parent training with this age group, but, linking to the pilot study *Methods* and/or *Results* sections, no data is presented to evaluate fidelity of treatment by the parents, a critical issue in evaluating child outcomes in a parent training based study. This is a structural flaw in the pilot study as it is presented for publication at present that precludes valid evaluation of this pilot study of child outcome variables. As such, it seem premature to publish this early result of child behaviors with no in-depth treatment of the parent training variable.

*Methods/Results*

 The four participants in the intervention study and the one control child are well described on relevant variables for inclusion and exclusion in the study. However, there is no description at all for the typical developmental comparison cohort. The only mentions I find are as follows:

1. on page 7, paragraph 2  in the *Results* section under MBL (……” typical control children without CG (12,15,16,18, and 20 months, listed in Morris, 2010”) 
2. under SSL, paragraph 1, “For ages 15 and 19 months, the only ages for which data were available for the treatment cohort and the typical children without CG.”

As a result, we have no comparable inclusionary and exclusionary information presented for the typically developing child group that is one of the three main comparison groups in the authors’ descriptive results graphs. As noted previously, there are no descriptions of data on parent training fidelity, which could potentially have a great impact on the child outcomes the authors report here. An unknown amount of the variability in results of this pilot study could be related to parent input issues related to the fidelity of delivery of intervention dimensions. Again, the pilot results on child outcome variables seems premature without this important dimension of the overall study of intervention delivered via parent interactions with the child. The *Limitations and future directions* gives a good road map for issues I would like to see available to a reader in this pilot study.

 Overall, this is a needed type of research in our field and has a fairly well developed basis in studies of parent training regimes that are available for comparison. However, no review of that dimension is present in this study. In addition, no description of the typically developing cohort used for comparison is available. I have given more detail in the original review related to these issues. I feel like the pilot study report would be better served as a picture of the larger study if all the dimensions of importance are equally well represented for the reader.

 Status: Major issues are missing to give the reader a full picture of the study contemplated and to fully evaluate the data results presented in this pilot study.

Is the work clearly and accurately presented and does it cite the current literature?

Partly

If applicable, is the statistical analysis and its interpretation appropriate?

No

Are all the source data underlying the results available to ensure full reproducibility?

No

Is the study design appropriate and is the work technically sound?

Partly

Are the conclusions drawn adequately supported by the results?

No

Are sufficient details of methods and analysis provided to allow replication by others?

Partly

Reviewer Expertise:

Early periods of child speech acquisition in typically developing and at-risk populations.

I confirm that I have read this submission and believe that I have an appropriate level of expertise to confirm that it is of an acceptable scientific standard, however I have significant reservations, as outlined above.

# Peer review: Reviewer response for version 1

**DOI:** 10.5256/f1000research.19753.r47831

**Published:** 2019-05-31

**Authors:** Barbara L. Davis

**Affiliations:** 1 Department of Communication Sciences and Disorders, University of Texas at Austin, Austin, TX, USA

*Summary:* This study reports on results of parent training for facilitating speech and language development for four young children with galactosemia and one control child whose parents were not trained in stimulation techniques. Outcome measures were compared with "typical control children" but the origin of that data was not described.   

*Is the work clearly and accurately presented and does it cite the current literature?*

 Citation about speech sound development is based on group studies of chronologically older children. No citations about variability in the pace of development. Would have been appropriate as the outcomes for these children are not supportive of the study rationale and could be due to variability either in diverse implementation by parents of the activities taught via telepractice, or diverse types of potential individual differences in the children. No literature review of parent training techniques, the major implementation method used.

 Child outcome measures carefully described. No description of outcomes of parent training provided. Those might help to understand data outcomes on child variables measured. 

*Is the study design appropriate and is the work technically sound?*

 In this developmental period, parent training is a well established way to conduct intervention. Some outcome variables focused on intervention fidelity and range of potential differences across parents would be appropriate . 

*Are sufficient details of methods and analysis provided to allow replication by others?*

 Very good information on child measurements with respected analyses or tests. No information about parent input analyses. I do not believe they are available.

 I *f applicable, is the statistical analysis and its interpretation appropriate?*

 Not applicable for this pilot study.

*Are all the source data underlying the results available to ensure full reproducibility?*

 Raw data not available but types of analyses are standards in the field. This aspect of the study is well described.

*Are the conclusions drawn adequately supported by the results?*

 In this pilot cohort of four CG children, one control, and and data from a group of comparison TD children, results do not support assertions made in Discussion. No measure of parent behaviors, the method of implementation for BBC intervention method. Either diverse implementation by parents of the activities they were taught via telepractice, or diverse types of potential individual differences in the children could account for the findings in the pilot data on MBL and SSL the primary issue of interest in measuring BBC intervention outcomes. I am very supportive of the importance of intervention research and focus on early intervention in ‘at risk’ populations. Unfortunately, the number of unknowns that are not yet accounted for in this pilot study of four children and a control suggests that it may be a premature to make assertions about effects of BBC parent intervention with CG children based on this dataset.

Is the work clearly and accurately presented and does it cite the current literature?

Partly

If applicable, is the statistical analysis and its interpretation appropriate?

No

Are all the source data underlying the results available to ensure full reproducibility?

No

Is the study design appropriate and is the work technically sound?

Partly

Are the conclusions drawn adequately supported by the results?

No

Are sufficient details of methods and analysis provided to allow replication by others?

Partly

Reviewer Expertise:

Early periods of child speech acquisition in typically developing and at-risk populations.

I confirm that I have read this submission and believe that I have an appropriate level of expertise to confirm that it is of an acceptable scientific standard, however I have significant reservations, as outlined above.

# Peer review: Reviewer response for version 1

**DOI:** 10.5256/f1000research.19753.r46124

**Published:** 2019-05-01

**Authors:** Shelley Velleman, Claudia Abbiati

**Affiliations:** 1 Department of Communication Sciences and Disorders, University of Vermont, Burlington, VT, USA; 2 Interdisciplinary PhD Program in Human Functioning and Rehabilitation Science, University of Vermont, Burlington, VT, USA

This manuscript focuses on an important and relevant issue: determining whether proactive intervention strategies are beneficial for infants at risk for later speech or language deficits associated with a specific condition (Classic Galactosemia, henceforth “CG”). This is an ambitious longitudinal project. In this manuscript, the authors report on preliminary findings and directions for research.

**Is the work clearly and accurately presented and does it cite the current literature?**

 The primary limitation of the work in its current form is a lack of specificity. First, the literature review fails to give the reader a full sense of the related research or of the related clinical services that have gone before. For example, the authors state that, “very early speech and language services are not yet available” (p. 3). They add that, “The question is whether proactive, preventive treatment, if it existed, could reduce the deleterious effects associated with the disease [that results in risk for speech-language delay] …and thereby improve outcomes” (p. 3). These statements seriously downplay decades of research and of speech-language services in early intervention. As long ago as 1983, Tomasello and Todd ^1^ showed that levels of parental joint attention impacted their infants’ vocabularies between 12-18 months. Roberts and Kaiser’s systematic review ^2^ documents the research demonstrating that training parents to interact with their children in certain ways can impact linguistic outcomes. For example, more recently, Roberts *et al*. ^3^ trained caregivers to use enhanced milieu teaching using a “teach-model-coach-review” approach. This included not only telling and showing the caregivers what to do, but also coaching them as they tried out the strategies and reviewing their success afterwards. Treatment fidelity on the part of the trainers and on the part of the caregivers were both measured. These authors demonstrated that the frequency and accuracy with which the caregivers carried out the strategies that they were taught had an impact on the children’s communication progress. These are but a few of many studies documenting the value of SLPs training parents to interact with their infants and toddlers in a manner that will facilitate communication development, with the result that SLPs involved in early intervention typically do train parents to use these strategies. For example, ASHA’s ^4^ practice portal document on early intervention states that, “Services that include opportunities for families and caregivers to directly participate in intervention are essential to strengthen existing knowledge and skills and to promote the development of new abilities that enhance child and family outcomes” ^5^ and that, “SLPs often coach families, caregivers, and other team members in how to implement functional, language-enhancing strategies during daily activities (Brown, 2016 ^6^; Brown & Woods, 2015 ^7^, 2016 ^8^; Roberts & Kaiser, 2011 ^2^; Roberts, Kaiser, Wolfe, Bryant, & Spidalieri, 2014 ^3^; Searcy, 2011 ^9^; Shelden & Rush, 2010 ^10^).”

 Therefore, the authors need to specify how their study is similar to and different from what’s been done before. Unique and important characteristics of their study might include the focus on stimulating prelinguistic vocalizations specifically (versus communication/cognition more generally), beginning at such a young age, and intervention for children with this specific syndrome. However, the very brief descriptions that they provide of their caregiver trainings make it impossible to judge exactly what they are training the parents to do, how they are training them to do it, and whether or not the treatment fidelity of the trainers or of the parents are actually measured. In this sense, the work is not clearly presented. See below for further discussion of the current lack of specificity in these areas.

**Is the study design appropriate and is the work technically sound?**

 The authors are gathering specific data systematically from several children, selected according to specific criteria, including two control participants who are not receiving treatment (one of whom has CG and one of whom does not), across time. However, the authors need to explicitly state the study’s design at the beginning of methods section of this manuscript. Further, the work and its context within its content area and the methods used are not presented specifically enough to judge the soundness of the approach or the appropriateness of the conclusions that are drawn from the results. 

**Are sufficient details of methods and analysis provided to allow replication by others?**

 The authors do not provide adequate details about the participants and methods used in this study, specifically:

- Inclusion and exclusion criteria should be organized separately on page 4 for clarity. In addition to participant criteria, authors should report participant characteristics (i.e., hearing/vision status, whether they are receiving other interventions concurrently, family composition/status, whether participants stay at home or are enrolled in daycare, etc.). This information is important to include for later analyses, as these variables may be confounders, mediators and moderators. 
- In addition to participant information, characteristics need to be collected and provided about legal guardian(s), specifically (a) socioeconomic status, (b) level of education, and (c) estimated amount of time spent with participants (an important consideration for intervention implementation/ feasibility). The authors mention on page 4 that enrichment variables are monitored, but preliminary findings of how these variables may be influencing outcomes is important to report, even qualitatively. Again, this information is important to include for later analyses, as these variables may be confounders, mediators and moderators. 
- Frequency of parent training and consultation is reported as “once per week for approximately 10 minutes” (p. 4). There is no information on the fidelity of parent implementation of BBC strategies, nor is there information on what types of training are provided. For example, were parents just given information or did trainers observe them using strategies and offer feedback? Roberts *et al*. ^3^ recommend a four-step caregiver training process; Akamoglu and Dinnebeil ^11^ recommend six steps. Were recommendations such as these followed? Were the parents’ goals for their children taken into account? Brown ^6^ reports that, “Intervention studies using...a transactional [as opposed to uni-directional] coaching approach have shown to have positive effects on parent and child outcomes while aligning with the intent of family capacity-building (Brown & Woods, 2015 ^7^, 2016 ^8^; Roberts *et al*., 2014 ^3^; Wetherby et al., 2014 ^12^)” (p. 144). If fidelity measures were performed, how was it measured and what are the results? This is important for replication purposes and also for judging factors that may impact the results of the study. Roberts *et al*. ^3^ report that, despite the intensive attention that was paid in their study to telling, showing, coaching, and reviewing the intervention strategies that they taught to parents, “generalization of strategy use to the home varied by caregiver and by strategy” (p. 1864) and also by activity type (play, book-reading, etc.). 
- The fact that only approximately 10 minutes of training was provided weekly is problematic for clinical translation purposes, as this amount and frequency of SLP intervention is not billable or practical. 
- The authors report that 50 utterances are compiled and transcribed using the International Phonetic Alphabet; however, no information is provided about how this information is acquired or under what circumstances. What level of IPA transcription is used - broad or narrow? Is IPA used for precanonical babble (which is not appropriate)? If not, are precanonical utterances omitted from the calculations? Are parents instructed to interact with their children during the samples using instructed strategies or with specific toys/stimuli? Are other communication partners involved, such as siblings? 
- The authors deemed the participant pool insufficient for running statistical tests; however, more information needs to be provided qualitatively about the participants described in this pilot study.
- In terms of “quality control” on page 5, the authors say that scores were double-scored. The authors need to clarify whether they mean intra-rater or inter-rater reliability was performed. Rationale should also be provided as to why 15% of the data was selected for this type of reliability analysis. 

**Are all the source data underlying the results available to ensure full reproducibility?**

- The use of symbols to mark data across time points in Figures 1-4 is problematic, as data points overlap in scores. This makes it difficult to determine whether two participants had the same score at one point or whether scores were not determined at particular time points. The authors should modify these figures using lines to present trends instead. If data are missing, then this needs to be indicated/reported, similar to what is reported if a participant is enrolled after 12 months of age. 
- Since the primary outcome variables are speech sound production in babble, it would be advantageous for the authors to report information on the types and percentages of consonants and vowels that are acquired by the participants based on each 50 utterance sample compiled across time points. The same is also true about expressive language, specifically total number of words, word types, and stage 1 word types (i.e., operations of reference, semantic relations). These measures are valuable in monitoring progress and change over time, and are relevant based on the primary outcome variables of interest. 

**Are the conclusions drawn adequately supported by the results?**

- The authors draw only tentative conclusions regarding why the children in the study had various outcomes, such as, “Whether these patterns reflect global levels of disease severity, global deficits from the treatment, or a combination of these cannot be ascertained based on the present data” (p. 8). This is appropriate, given the currently small sample size. However, as noted above, the authors should provide much more information about other factors (i.e., parental and environmental characteristics) that are likely to have influenced the outcomes of the children reported in this preliminary account. 
- The authors state on page 5 that “Continued data collection and analysis in this population will provide conclusive evidence.” Given that the data reported are preliminary, this comment is appropriate. However, future evidence still should not be deemed as “conclusive,” but rather as “more promising”.
- It is too soon for the authors to draw conclusions about the validity of their measures based on these preliminary data. Therefore, their statement on page 7 that: “It is possible that MBL and SSL do not measure crucial weaknesses in children with CG in general” seems inappropriate at this time.
- The authors should omit or reword the phrase “Given the 90% risk for language impairment in children with CG” on page 8. This is a misinterpretation of the research reported by the authors on page 3: “language impairment in 90% of children with CG.” Risk should be reported as a statistical measure and cannot be inferred based on this reported proportion.

We look forward to further information and updates on this interesting study.

Is the work clearly and accurately presented and does it cite the current literature?

Partly

If applicable, is the statistical analysis and its interpretation appropriate?

Not applicable

Are all the source data underlying the results available to ensure full reproducibility?

No

Is the study design appropriate and is the work technically sound?

Partly

Are the conclusions drawn adequately supported by the results?

Partly

Are sufficient details of methods and analysis provided to allow replication by others?

No

Reviewer Expertise:

Infant, toddler, and child speech development and disorders, including prelinguistic vocalizations and early words, and motor speech development and disorders.

We confirm that we have read this submission and believe that we have an appropriate level of expertise to confirm that it is of an acceptable scientific standard, however we have significant reservations, as outlined above.

## References

[1] : Joint attention and lexical acquisition style. *First Language*.1983;4(12) : 10.1177/014272378300401202 197-211 10.1177/014272378300401202

[2] : The Effectiveness of Parent-Implemented Language Interventions: A Meta-Analysis. *American Journal of Speech-Language Pathology*.2011;20(3) : 10.1044/1058-0360(2011/10-0055) 180-199 10.1044/1058-0360(2011/10-0055) 21478280

[3] : Effects of the teach-model-coach-review instructional approach on caregiver use of language support strategies and children's expressive language skills. *J Speech Lang Hear Res*.2014;57(5) : 10.1044/2014_JSLHR-L-13-0113 1851-69 10.1044/2014_JSLHR-L-13-0113 24950492

[4] : Early Intervention (Practice Portal). *[accessed 28th April 2019]*. Reference source

[5] : DEC recommended practices in early intervention/early childhood special education 2014. *[accessed 28th April 2019]*. Reference source

[6] : Coaching in Parent-Implemented Early Communication Interventions: Understanding and Overcoming Individual-Level Implementation Barriers. *Perspectives of the ASHA Special Interest Groups*.2016;1(1) : 10.1044/persp1.SIG1.144 144-153 10.1044/persp1.SIG1.144

[7] : Effects of a Triadic Parent-Implemented Home-Based Communication Intervention for Toddlers. *Journal of Early Intervention*.2015;37(1) : 10.1177/1053815115589350 44-68 10.1177/1053815115589350

[8] : Parent-Implemented Communication Intervention. *Topics in Early Childhood Special Education*.2016;36(2) : 10.1177/0271121416628200 115-124 10.1177/0271121416628200

[9] : Here's how to do early intervention for speech and language: Empowering parents. *Plural Publishing*.2012;

[10] : A primary-coach approach to teaming and supporting families in early childhood intervention. *Working with families of young children with special needs*.2010;175-202

[11] : Coaching parents to use naturalistic language and communication strategies. *Young Exceptional Children*.2017;20:41-50

[12] : Parent-implemented social intervention for toddlers with autism: an RCT. *Pediatrics*.2014;134(6) : 10.1542/peds.2014-0757 1084-93 10.1542/peds.2014-0757 25367544PMC4243066
